# The mediating role of maternal metabolites between lipids and adverse pregnancy outcomes of gestational diabetes mellitus

**DOI:** 10.3389/fmed.2022.925602

**Published:** 2022-08-10

**Authors:** Mingjuan Luo, Jingyi Guo, Wenqian Lu, Xiangnan Fang, Rong Zhang, Mengyang Tang, Qiong Luo, Wei Liang, Xiangtian Yu, Cheng Hu

**Affiliations:** ^1^Department of Endocrinology and Metabolism, University of Hong Kong-Shenzhen Hospital, Shenzhen, China; ^2^The Third School of Clinical Medicine, Southern Medical University, Guangzhou, China; ^3^Department of Endocrinology and Metabolism, Fengxian Central Hospital Affiliated to the Southern Medical University, Shanghai, China; ^4^Clinical Research Center, Shanghai Jiao Tong University Affiliated Sixth People's Hospital, Shanghai, China; ^5^Department of Endocrinology, First Affiliated Hospital of Gannan Medical University, Ganzhou, China; ^6^Shanghai Diabetes Institute, Shanghai Jiao Tong University Affiliated Sixth People's Hospital, Shanghai, China; ^7^Department of Obstetrics and Gynecology, University of Hong Kong-Shenzhen Hospital, Shenzhen, China

**Keywords:** gestational diabetes mellitus, maternal metabolism, adverse pregnancy outcomes, lipids, mediation analysis

## Abstract

Gestational diabetes mellitus (GDM) is one of the most common complications of pregnancy, and the demographics of pregnant women have changed in recent decades. GDM is a metabolic disease with short- and long-term adverse effects on both pregnant women and newborns. The metabolic changes and corresponding risk factors should be of great significance in understanding the pathological mechanism of GDM and reducing the incidence of adverse pregnancy outcomes in patients with GDM. The well-known GDM-associated lipids used in clinical tests, such as triglyceride (TG), are thought to play a major role in metabolic changes during GDM, which have a potential causal relationship with abnormal pregnancy outcomes of GDM. Therefore, this study analyzed the relationship between clinical lipid indicators, metabolic profiles, and abnormal pregnancy outcomes in GDM through mediation analysis. By constructing a metabolic atlas of 399 samples from GDM patients in different trimesters, we efficiently detected the key metabolites of adverse pregnancy outcomes and their mediating roles in bridging abnormal lipids and adverse pregnancy outcomes in patients with GDM. Our study confirmed that TG and total cholesterol were independent risk factors for adverse pregnancy outcomes in patients with GDM. Several key metabolites as mediators (e.g., gamma-linolenic acid, heptadecanoic acid, oleic acid, palmitic acid, and palmitoleic acid) have been identified as potential biomarkers for adverse pregnancy outcomes in patients with GDM. These metabolites mainly participate in the biosynthesis of unsaturated fatty acids, which may shed new light on the pathology of GDM and provide insights for further exploration of the molecular mechanisms underlying adverse pregnancy outcomes.

## Introduction

Gestational diabetes mellitus (GDM) is one of the most common pregnancy-related complications and is defined as glucose intolerance that is first recognized during pregnancy (1, 2). The demographics of pregnant women have changed in recent decades, with older maternal age and rising obesity rates contributing to the increasing prevalence of GDM, making this disease a global problem (3). According to the International Association of Diabetes in Pregnancy Study Group (IADPSG) (4), the global prevalence of GDM in 2021 was 14.0% (95% confidence interval, CI: 13.97–14.04%) and the regional prevalence ranges from 7.1–27.6%. Specifically, the prevalence of GDM in Southeast Asia is the second highest in the world, reaching 20.8% (5).

GDM has both short- and long-term adverse effects on pregnant women and newborns. For example, women with GDM are more likely to develop polyhydramnios, preeclampsia, and preterm delivery than those with normal blood glucose levels. Poor maternal blood glucose control also increases the risk of macrosomia, malformations, and even stillbirth (6). In the long term, GDM can result in adverse metabolic sequelae in the mother and offspring. For example, studies have shown that 35–60% of women with GDM develop type 2 diabetes mellitus within 10–20 years of pregnancy (7, 8).

Risk factors for GDM include being overweight or obese, advanced maternal age, family history of diabetes, previous history of GDM, excessive weight gain during pregnancy, history of polycystic ovary syndrome, and habitual smoking (9). In addition to the recognized effects of hyperglycemia, other metabolic indicators may also be important for neonatal health (10). For example, previous studies have indicated that hyperlipidemia during pregnancy increases the incidence of GDM and preeclampsia (11, 12). A study conducted in a Chinese population showed that high triglyceride (TG) concentrations in pregnant women during the third trimester of pregnancy were independently and significantly associated with an increased risk of GDM, preeclampsia, intrahepatic cholestasis of pregnancy, large for gestational age (LGA), macrosomia, and reduced risk of small for gestational age (SGA). Additionally, the high-density lipoprotein cholesterol (HDL-C) level was negatively correlated with the risk of GDM and macrosomia, and positively correlated with the risk of SGA. However, no correlations were observed between low-density lipoprotein cholesterol (LDL-C) or total cholesterol (TC) levels and adverse neonatal pregnancy outcomes (13). Another study in China showed that TC levels during the third trimester of pregnancy were associated with a lower risk of SGA, whereas HDL-C and LDL-C levels during late pregnancy were associated with an increased risk of SGA (14). Several studies have shown that in well-controlled GDM, maternal TG concentration is positively correlated with newborn fat mass and LGA (15, 16).

Therefore, the metabolic changes and corresponding risk factors should be the focus in understanding the pathological mechanism of GDM and reducing the incidence of adverse pregnancy outcomes in patients with GDM. Currently, metabolic molecular markers provide a new perspective for disease prediction and diagnosis. Moreover, metabolomics has been widely used in the study of metabolic diseases to detect the changes of metabolites caused by pathophysiological changes. Indeed, metabolomics is the ultimate downstream product of gene transcription; therefore, it could also reflect the epigenetic and genetic interactions involved in the progression of GDM, which could help develop new biomarkers for GDM.

Most studies have focused on the metabolic changes in pregnant women with GDM. For example, Hou et al. (17) used ultra-performance liquid chromatography-mass spectrometry (UPLC-MS), gas chromatography, and nuclear magnetic resonance to detect maternal serum (in 131 GDM and 138 control cases) and found that free fatty acids, branched-chain amino acids, lipids, and organic oxygen compounds showed significant changes, which differed in the control and GDM groups. However, few metabolomics studies have predicted the adverse pregnancy outcomes of GDM.

Identifying serum metabolic molecules in the second or third trimester of pregnancy as biomarkers of adverse postpartum outcomes in patients with GDM is crucial. In particular, the well-known GDM-associated lipids in clinical tests and their potential causal relationship with abnormal pregnancy outcomes of GDM are thought to play a major role in metabolic changes during GDM. Therefore, we used mediation analysis to explore the relationship between clinical lipid indicators, metabolic profiles, and abnormal pregnancy outcomes in GDM. We constructed a metabolic atlas of GDM using UPLC-MS on 399 samples from patients with GDM in different trimesters. We also detected key metabolites discriminating adverse pregnancy outcomes and their mediating roles in bridging abnormal lipids and adverse pregnancy outcomes in patients with GDM. It may shed new light on the pathology of GDM and provide insights for further exploration of the molecular mechanisms underlying adverse pregnancy outcomes.

## Materials and methods

### Ethical approval of the study protocol

The study protocol was approved by the Ethics Committee of the University of Hong Kong-Shenzhen Hospital ([2014] 98, [2017] 13). Written informed consent was obtained from all participants. Personal data were anonymized and omitted.

### Study enrollment and adverse pregnancy outcome evaluation

All samples were taken from women with GDM, 200 in the second trimester and 199 in the third trimester, at the University of Hong Kong-Shenzhen Hospital from 2015 to 2018. Participants with cardiovascular, cerebrovascular, liver, kidney, and autoimmune diseases, malignant tumors, and long-term use of glucocorticoids and other drugs affecting glucose and lipid metabolism were excluded. The diagnostic criteria for GDM were based on the IADPSG criteria. Pregnant women took 75 g of glucose between 24–28 weeks of gestation, and their venous blood glucose was measured at fasting, 1 and 2 h after glucose administration. GDM was diagnosed if fasting blood glucose was ≥5.1 mmol/L, 1 h blood glucose ≥10.0 mmol/L, or 2 h blood glucose ≥8.5 mmol/L.

Adverse neonatal outcomes included fetal distress, preterm birth, small for gestational age (SGA), macrosomia, hyperbilirubinemia, neonatal malformation, and stillbirth. Macrosomia was defined as a birth weight ≥4,000 g. Preterm birth was defined as those whose gestational age was <37 weeks. Finally, SGA referred to infants whose birth weight was below the 10th percentile of the average weight at the same gestational age. The participants were divided into two groups: an adverse pregnancy outcome group (A group, *n* = 48) and a normal pregnancy outcome group (N group, *n* = 351). In A group, fetal distress (*n* = 9), preterm birth (*n* = 20), SGA (*n* = 7), macrosomia (*n* = 16), hyperbilirubinemia (*n* = 14), neonatal malformation (*n* = 2), and stillbirth (*n* = 1) were included.

### Sample collection and serum metabolomics measurements

Clinical data, such as age, height, pre-pregnancy weight, pregnancy weight gain, and family history of diabetes were collected for every participant, and indicators such as glycosylated hemoglobin (HbA1c) in early pregnancy, blood lipid, and total bile acid levels in late pregnancy were recorded. Information on pregnancy outcomes was obtained from the hospital's maternal and infant medical records. Body mass index (BMI) was calculated as mass (kg) divided by height in meters squared (m^2^). Serum samples were obtained at 24 weeks of gestation or after. Blood samples were collected in the morning after an overnight fast through the anterior cubital vein. Blood glucose and total bile acid levels were determined by hexokinase and cyclic enzymes, respectively, using a Cobas 8000 biochemical analyzer (Roche Ltd., Basel, Switzerland). Blood lipids, including TC, LDL-C, HDL-C, and TG were measured using spectrophotometry on a Siemens ADVIA-2400 automatic biochemical analyzer (Siemens AG, Munich, Germany). HbA1c was determined by high-pressure liquid chromatography (HPLC) using an HbA1c HA-8160 analyzer (Arkray Ltd., Kyoto, Japan).

Serum samples from each participant were collected and stored at −80°C. Metabolomic analysis was performed using a metabO-Profile (Shanghai, China) (18) as bellows.

For targeted metabolites, all their standards were accurately prepared and weighed in methanol or water. The individual stock solution was obtained with a concentration of 5 mg/mL, and appropriate amount of each stock solution was mixed for stock calibration solutions. Meanwhile, a mixture of stable isotope labeled internal standards was prepared in methanol at a concentration of 50 μM/L.

25 μL of serum was added to a 96-well-plate, which was transferred to the Biomek 4000 automation workstation (Biomek 4000, Beckman Coulter, Inc., California, USA). For extracting the metabolites, ~120 μL of ice-cold methanol with partial internal standards was added to each sample automatically. After vortexing for 5 min, the mixture was centrifuged for 30 mins at 4,000 g; 30 μL of supernatant was then transferred to a new 96-well-plate, and 20 μL of freshly prepared derivative reagents (3-Nitrophenylhydrazine) was added to each well. After derivatization for 60 min at 30°C, 330 μL of ice-cold 50% methanol solution was added for dilution. The samples were stored at −20°C for 20 mins and were centrifuged at 4,000 g for 30 mins at 4°C. Approximately 135 μL of supernatant was mixed with 10 μL of internal standards in each well of a new 96-well-plate. Serial dilutions of derivatized stock standards were added to the left wells, and the plate was ready for analysis.

Chromatographic separation was performed on an ACQUITY UPLC BEH C18 VanGuard pre-column (2.1 separation was performed on an ACQUITY UPLC BEH C18 VanGuarde reagents (3-Nitrophenylhydrazine) was added to each we the sample manager was set at 40 and 10°C, respectively. The mobile phase A was water with 0.1% formic acid, while B was a mixture of acetonitrile and isopropanol (70:30). Gradient conditions were 0–1 min, 5% B; 1–11 min, 5–78% B; 11–13.5 min, 78–95% B; 13.5–14 min, 95–100% B; 14–16 min, 100% B; 16–16.1 min, 100–5% B; 16.1–18 min, 5% B. The flow rate was 0.4 mL/min with a 5 μL injection volume.

The mass spectrometer was operated in positive electrospray ionization (ESI+) mode with a capillary voltage of 1.5 kV as well as the negative electrospray ionization (ESI–) mode with a capillary voltage of 2 kV. The temperature of the ion source and desolvation was 150 and 550°C, respectively. The desolvation gas flow was set at 1,000 L/h. Raw data files generated by UPLC-MS/MS were processed using Masslynx software (v4.1, Waters, Milford, MA, USA), which can perform peak integration, calibration, and quantitation for each metabolite.

LOD (limit of detection) (19) was applied to fill in missing values of quantitative metabolomic data. Due to above metabolomic analysis was an absolute quantification rather than non-targeted relative quantification, no standardization is required in this step here.

### Statistic analyses of clinical characteristics

The participants were divided into groups A and N. Continuous characteristic variables are expressed as the mean ± standard deviation or median (interquartile range), according to the results of normality testing. Categorical variables are expressed as proportions. Clinical characteristics between groups were determined using Student's *t*-test or Wilcoxon test for continuous variables and Pearson's chi-squared test for categorical variables. All statistical analyses were performed using R version 4.1.3 (https://www.r-project.org/). Statistical significance was defined as a two-sided *P* < 0.05.

We performed a *post-hoc* power calculation on the GDM cohort using PASS 15 Power Analysis and Sample Size Software (NCSS, LLC. Kaysville, Utah, USA). The cohort was divided into a high TG group (≥1.7 mmol/L, *n* = 336) and a normal TG group (<1.7 mmol/L, *n* = 63) based on serum triglyceride levels, with an incidence of adverse pregnancy outcomes of 13.39% (45/336) and 4.76% (3/63), respectively. The statistical power of the *post-hoc* analysis to detect differences between the two groups was 75.33%.

### Discrimination analysis of effect of metabolite communities on GDM adverse pregnancy outcomes

To observe the differences in metabolite abundance between the two groups, we used MetaboAnalyst 5.0 (20) (https://www.metaboanalyst.ca), a web-based tool for analyzing metabolomic data. Univariate and multivariate analyses included differential expression analysis using *t-*tests, fold-change with volcano plot, principal component analysis (PCA), and orthogonal partial least square discriminant analysis (OPLS-DA). Data processing was performed using the iMAP platform (version 1.0, Metabo-Profile, Shanghai, China). In univariate analysis, *P* < 0.05 and |log2FC| > 0 were used to determine differentially expressed metabolites (DEMs). To investigate the biological function of the DEMs, enrichment and pathway analyses were performed with metabolite set enrichment analysis (MSEA). Functions or pathways were considered impactful with a false discovery rate (FDR) <0.05.

### Mediation analysis of metabolites for causal relationship of serum lipids with adverse pregnancy outcome

We used univariate logistic regression analysis to evaluate the independent effect of four serum lipids: TG, TC, HDL-C, and LDL-C on the adverse pregnancy outcomes of GDM. Multiple logistic regression analysis was further adopted to adjust for potential confounders, including age, pre-gestational BMI, changes in BMI during pregnancy, and family history of diabetes.

Non-conditional logistic regression was performed to uncover the relationship between adverse pregnancy outcomes and metabolic features at *P* < 0.05. As risk factors for adverse pregnancy outcomes, we further explored the association of these significant metabolites with TG and TC levels using general linear regression. All metabolite concentrations were log-transformed before analysis to obtain an approximately normal distribution. We reported the odds ratios (OR) and beta coefficients (β) with 95% CI and *P*-values.

Finally, we conducted a causal mediation analysis to assess whether metabolites could be potential mediators linking serum lipids to adverse pregnancy outcomes, using the R “mediation” package (21) adjusted for age, pregestational BMI, changes of BMI during pregnancy, and family history of diabetes. In this model, the total effect was separated into average direct effects (ADEs) and average causal mediation effects (ACMEs). Additionally, the mediated proportion indicated how much of the total effect can be explained by the ACME.

In order to assess the composite association of metabolites with significant mediator effects, metabolite scores for adverse pregnancy outcomes were calculated as the weighted sum of these metabolites' levels. The weight of each metabolite was the regression coefficient for a 1-standard deviation increment in the serum metabolite levels estimated by the multivariable logistic regression model. The metabolite scores were also analyzed for mediation effects.

To better understand the biological function of the key metabolites selected by mediation analysis, co-expression analysis of the correlation network was performed against these metabolic markers to capture neighboring communities whose members have similar metabolic expression patterns as key metabolites. Such key metabolic marker-related communities were made up of the top 25 correlated metabolites, making up a functional group whose enriched pathways can be analyzed with MSEA. Function or pathway enrichment was considered significant with FDR <0.05.

## Results

### Clinical characteristics of participants

Patients with GDM were screened according to the inclusion and exclusion criteria at the University of Hong Kong-Shenzhen Hospital. A total of 399 patients with GDM from January 1, 2015 to September 1, 2018 participated in this study. Serum was collected from each patient and processed using UPLC-MS/MS for metabolomics analysis. The clinical characteristics of each participant are presented in Table 1. Compared with the N group, those in the A group were older (*P* = 0.023) and had higher TC levels (*P* = 0.038). BMI, family history of diabetes, HbA1c, TG, HDL-C, LDL-C, TBA, fasting blood glucose (FBG), and 1 h and 2 h blood glucose levels after conducting oral glucose tolerance test (OGTT) were similar between the two groups.

**Table 1:** Characteristics of adverse and normal pregnancy outcome groups.

	**Normal pregnancy** **outcome group** **(*n* = 351)**	**Adverse pregnancy** **outcome group** **(*n* = 48)**	***P***
Age (years)	29 (28, 31)	30 (29, 33)	0.023^*^
Pre-gestational BMI (kg/m^2^)	21.05 ± 2.60	21.26 ± 2.71	0.604
Changes in BMI (kg/m^2^)	4.63 ± 1.56	4.81 ± 1.41	0.461
FBG (mmol/L)	4.59 ± 0.36	4.59 ± 0.34	0.906
1 h-PG (mmol/L)	9.82 ± 1.38	9.70 ± 1.79	0.612
2 h-PG (mmol/L)	8.64 ± 1.43	8.68 ± 1.68	0.863
HbA1c (%)	5.19 ± 0.25	5.14 ± 0.32	0.175
Total cholesterol (mmol/L)	5.80 (5.10, 6.60)	6.16 (5.43, 7.52)	0.038^*^
Triglycerides (mmol/L)	2.29 (1.87, 2.96)	2.34 (1.94, 4.06)	0.076
LDL-C (mmol/L)	3.21 (2.59, 3.85)	3.59 (2.60, 4.23)	0.094
HDL-C (mmol/L)	2.00 ± 0.41	1.90 ± 0.38	0.117
TBA (μmol/L)	2.20 (1.57, 3.05)	2.14 (1.52, 2.44)	0.415
Family history of diabetes, *n* (%)	87 (24.8)	16 (33.3)	0.204

*BMI, body mass index; FBG, fasting blood glucose; 1 h-PG, one hour postprandial glucose; 2 h-PG, two hours postprandial glucose; LDL-C, low-density lipoprotein cholesterol; HDL-C, high-density lipoprotein cholesterol; TBA, total bile acid*.

* *P < 0.05*.

The risk of adverse pregnancy outcomes was directly correlated with TG (OR = 8.034, 95% CI = 1.548–41.694, *P* = 0.013) and TC levels (OR = 87.500, 95% CI = 2.040–3,752.335, *P* = 0.020). This positive association was further validated when the models were adjusted for covariates (OR = 5.90, 95% CI = 1.081–32.210, *P* = 0.040 for TG; OR = 58.68, 95% CI = 1.222–2,818.823, *P* = 0.039 for TC) (Table 2). No association was observed between HDL-C or LDL-C levels and adverse pregnancy outcomes, and the candidate metabolite mediators of causal relation between such clinical lipids and adverse pregnancy outcome were further investigated in this study.

**Table 2:** Association of serum lipids with adverse pregnancy outcome of GDM.

**Lipids**	**Crude OR (95% CI)**	***P***	**Adjusted OR (95% CI)^a^**	***P***
TG	8.034 (1.548–41.694)	0.013^*^	5.900 (1.081–32.210)	0.040^*^
TC	87.500 (2.040–3,752.335)	0.020^*^	58.681 (1.222–2,818.823)	0.039^*^
HDL-C	0.090 (0.004–2.219)	0.141	0.099 (0.004–2.491)	0.160
LDL-C	8.646 (0.709–105.474)	0.091	6.731 (0.537–84.435)	0.140

a *Adjusted for age, pre-gestational BMI, changes in BMI, and family history of diabetes*.

*OR, odds ratio; CI, confidence interval; TG, triglycerides; TC, total cholesterol; HDL-C, high-density lipoprotein cholesterol; LDL-C, low-density lipoprotein cholesterol*.

* *P < 0.05*.

### Metabolomics profiling of GDM associated with different pregnancy outcome

**Figure 1:**
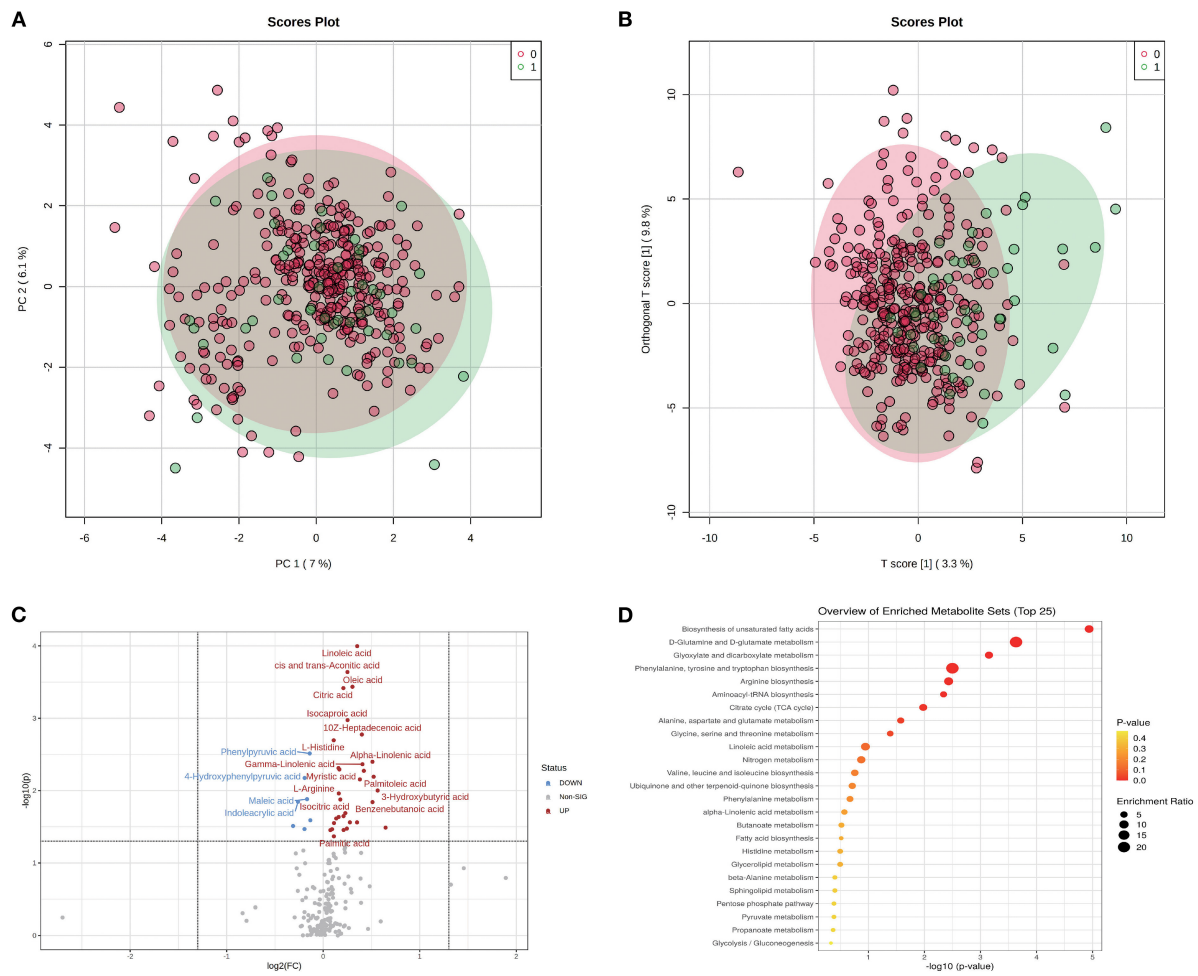
Metabolomic profiles of GDM and discrimination analysis of adverse pregnancy outcome. **(A)** Sample distribution in PCA model for GDM patients in N group (annotated as 0) and A group (annotated as 1). **(B)** Sample distribution in OPLS-DA model for GDM patients in N group (annotated as 0) and A group (annotated as 1). **(C)** DEMs between N and A group evaluated in volcano plot. **(D)** The functional enrichment of DEMs in KEGG database.

On one hand, the association between metabolites and pregnancy outcome were validated by conventional differential expression analysis. As shown in Figure 1A, the PCA results revealed that the A group could not be simply separated from the N group by several PC components. Meanwhile, the results of OPLS-DA (Figure 1B) indicated the possibility of distinguishing the differences between the two groups using selected DEMs. We determined the DEMs using |log2FC| > 0 and *P* < 0.05 (whole FDR are listed in Supplementary Table 1). The volcano plot displays the significant differences in metabolite expression levels between the two groups (Figure 1C). As shown in the volcano plot, 38 of the 200 metabolites detected in this study (Supplementary Table 1) were identified as DEMs. Most DEMs were upregulated in the A group, and the top five DEMs were linoleic acid, cis- and trans-aconitic acid, oleic acid, citric acid, and isocaproic acid.

The dysfunctions in these DEMs were further evaluated using MSEA (Figure 1D). Biosynthesis of the unsaturated fatty acids pathway had the highest fold enrichment and lowest *p-*value. Other significantly enriched functions included D-glutamine and D-glutamate metabolism and glyoxylate and dicarboxylate metabolism pathways, which had an FDR <0.05 (Supplementary Table 2).

### Key metabolic markers as mediators of causal relation between clinical lipids and adverse pregnancy outcome

We performed a non-conditional logistic regression analysis to reveal the relationship between adverse pregnancy outcomes and metabolism indicators and found 40 metabolites associated with adverse pregnancy outcomes (*P* < 0.05) (Table 3). Among these metabolites, 22 and 31 were affected by TC and TG, respectively. Of the 40 metabolite mediators associated with adverse pregnancy outcomes, 38 were identical to above DEMs, except for glucaric acid and valeric acid, which suggest the high confidence of these key metabolites from different analysis methods.

**Table 3:** Metabolites associated with adverse pregnancy outcome at *P* < 0.05 and their associations with TG or TC.

**Metabolites**	**Adverse pregnancy outcomes**		**TG**		**TC**	
	**OR (95% CI)**	***P***	**β (95% CI)**	***P***	**β (95% CI)**	***P***
Adrenic acid	4.865 (1.183–20.015)	0.0283	0.37922 (0.26069–0.49774)	<0.0001^*^	0.41455 (0.16117–0.66793)	0.0014^*^
Alpha-Linolenic acid	5.150 (1.689–15.700)	0.0040	0.46223 (0.28688–0.63759)	<0.0001^*^	0.50266 (0.1318–0.87351)	0.008^*^
Arachidonic acid	7.595 (1.305–44.191)	0.0240	0.20832 (0.11758–0.29906)	<0.0001^*^	0.35899 (0.17015–0.54782)	0.0002^*^
Benzenebutanoic acid	1.904 (1.146–3.161)	0.0128	0.82729 (0.38723–1.26735)	0.0002^*^	0.7305 (−0.19092–1.65191)	0.1199
Caproic acid	5.100 (1.197–21.726)	0.0276	0.1646 (0.04965–0.27955)	0.0051^*^	0.1836 (−0.05545–0.42265)	0.1318
Citric acid	160.693 (9.108–>999.999)	0.0005	0.24881 (0.19111–0.30652)	<0.0001^*^	0.12871 (−0.00017–0.25758)	0.0503
Gamma-Linolenic acid	5.827 (1.734–19.577)	0.0044	0.39232 (0.23834–0.54629)	<0.0001^*^	0.481 (0.1566–0.80541)	0.0038^*^
Glucaric acid	14.468 (1.194–175.33)	0.0358	0.15949 (0.0242–0.29477)	0.021^*^	−0.12245 (−0.40344–0.15855)	0.3921
Glyceric acid	9.083 (1.150–71.746)	0.0364	0.13036 (0.04979–0.21093)	0.0016^*^	0.04826 (−0.12015–0.21668)	0.5735
Heptadecanoic acid	4.361 (1.193–15.936)	0.0259	0.26592 (0.06678–0.46505)	0.009^*^	0.54254 (0.13124–0.95385)	0.0099^*^
Indoleacrylic acid	0.518 (0.294–0.9110)	0.0224	−0.16371 (−0.39857–0.07114)	0.1713	0.55237 (0.06931–1.03544)	0.0251^*^
Isocaproic acid	30.226 (3.783–241.528)	0.0013	0.17987 (0.09735–0.26239)	<0.0001^*^	0.30283 (0.1311–0.47456)	0.0006^*^
Isocitric acid	17.835 (1.808–175.939)	0.0136	0.2675 (0.18946–0.34554)	<0.0001^*^	0.0113 (−0.15882–0.18143)	0.8962
L-Arginine	32.437 (2.177–483.395)	0.0116	0.16937 (0.105–0.23373)	<0.0001^*^	0.2948 (0.1606–0.42901)	<0.0001^*^
L-Glutamine	224.516 (1.427–>999.999)	0.0359	0.02757 (−0.00808–0.06322)	0.1292	0.0824 (0.00902–0.15579)	0.0278^*^
L-Histidine	>999.999 (11.514–>999.999)	0.0025	0.04761 (0.00954–0.08569)	0.0144^*^	0.09187 (0.01317–0.17057)	0.0223^*^
L-Homoserine	34.037 (1.546–749.346)	0.0253	0.08294 (0.02612–0.13976)	0.0043^*^	0.13324 (0.01544–0.25105)	0.0267^*^
L-Lactic acid	0.113 (0.016–0.780)	0.0270	−0.10157 (−0.18935– −0.01379)	0.0235^*^	−0.27098 (−0.451450– −0.0905)	0.0033^*^
L-Serine	70.303 (1.340–>999.999)	0.0353	0.08732 (0.04502–0.12962)	<0.0001^*^	0.12951 (0.04129–0.21773)	0.0041^*^
L-Threonine	51.869 (1.490–>999.999)	0.0293	0.07664 (0.02845–0.12483)	0.0019^*^	0.10332 (0.0031–0.20354)	0.0433^*^
Linoleic acid	29.491 (5.103–170.446)	0.0002	0.31197 (0.21229–0.41164)	<0.0001^*^	0.29382 (0.08036–0.50728)	0.0071^*^
Maleic acid	0.607 (0.387–0.953)	0.0300	−0.05321 (−0.3236–0.21717)	0.699	−0.1958 (−0.75393–0.36233)	0.4908
Myristic acid	6.041 (1.607–22.703)	0.0078	0.45158 (0.32975–0.57341)	<0.0001^*^	0.61044 (0.34942–0.87145)	<0.0001^*^
N-Acetylglycine	66.440 (3.351–>999.999)	0.0059	0.10103 (0.04868–0.15337)	0.0002^*^	0.08619 (−0.02352–0.1959)	0.1233
Oleic acid	33.346 (4.662–238.532)	0.0005	0.30158 (0.20812–0.39504)	<0.0001^*^	0.33076 (0.13086–0.53066)	0.0012^*^
Oxoglutaric acid	0.220 (0.054–0.896)	0.0345	−0.13701 (−0.26678– −0.00723)	0.0386^*^	−0.26952 (−0.53764– −0.0014)	0.0488^*^
Palmitic acid	16.987 (1.094–263.711)	0.0429	0.11252 (0.04455–0.18049)	0.0012^*^	0.22638 (0.08592–0.36684)	0.0017^*^
Palmitoleic acid	4.308 (1.628–11.396)	0.0033	0.49284 (0.24801–0.73766)	<0.0001^*^	0.73257 (0.22222–1.24292)	0.005^*^
Phenylpyruvic acid	0.485 (0.276–0.854)	0.0122	−0.0447 (−0.26109–0.1717)	0.6849	0.18061 (−0.266–0.62721)	0.4271
Propanoic acid	5.442 (1.278–23.178)	0.0219	0.24689 (0.13679–0.35698)	<0.0001^*^	0.2308 (−0.00093–0.46254)	0.0509
Ricinoleic acid	41.812 (3.168–551.921)	0.0046	0.11671 (0.04029–0.19314)	0.0028^*^	0.14203 (−0.01696–0.30102)	0.0798
Stearylcarnitine	0.620 (0.394–0.977)	0.0394	0.10107 (−0.18979–0.39193)	0.4949	0.48039 (−0.11872–1.0795)	0.1157
Taurochenodeoxycholic acid	2.299 (1.082–4.884)	0.0304	−0.15611 (−0.58007–0.26786)	0.4696	−0.16623 (−1.04215–0.7097)	0.7093
8,11,14-Eicosatrienoic acid	5.234 (1.619–16.929)	0.0057	0.47451 (0.33066–0.61837)	<0.0001^*^	0.60046 (0.29373–0.90719)	0.0001^*^
cis and trans-Aconitic acid	91.529 (7.689–>999.999)	0.0004	0.27292 (0.20771–0.33812)	<0.0001^*^	0.10345 (−0.04187–0.24878)	0.1624
Valeric acid	7.210 (1.396–37.228)	0.0184	−0.09279 (−0.33195–0.14636)	0.446	0.11303 (−0.38106–0.60713)	0.6531
10Z-Heptadecenoic acid	7.779 (2.137–28.324)	0.0019	0.42406 (0.27856–0.56957)	<0.0001^*^	0.61861 (0.31198–0.92525)	<0.0001^*^
3-Hydroxybutyric acid	3.178 (1.303–7.752)	0.0110	0.3072 (0.12169–0.49271)	0.0012^*^	0.13482 (−0.25313–0.52276)	0.4949
3-Hydroxyisovaleric acid	9.433 (1.375–64.705)	0.0224	0.03627 (−0.05705–0.1296)	0.4452	−0.03109 (−0.22392–0.16175)	0.7515
4-Hydroxyphenylpyruvic acid	0.602 (0.405–0.894)	0.0120	0.05965 (−0.26065–0.37994)	0.7145	0.51063 (−0.14899–1.17024)	0.1288

*TG, triglycerides; TC, total cholesterol; OR, odds ratio; CI, confidence interval*.

* *P < 0.05*.

**Figure 2:**
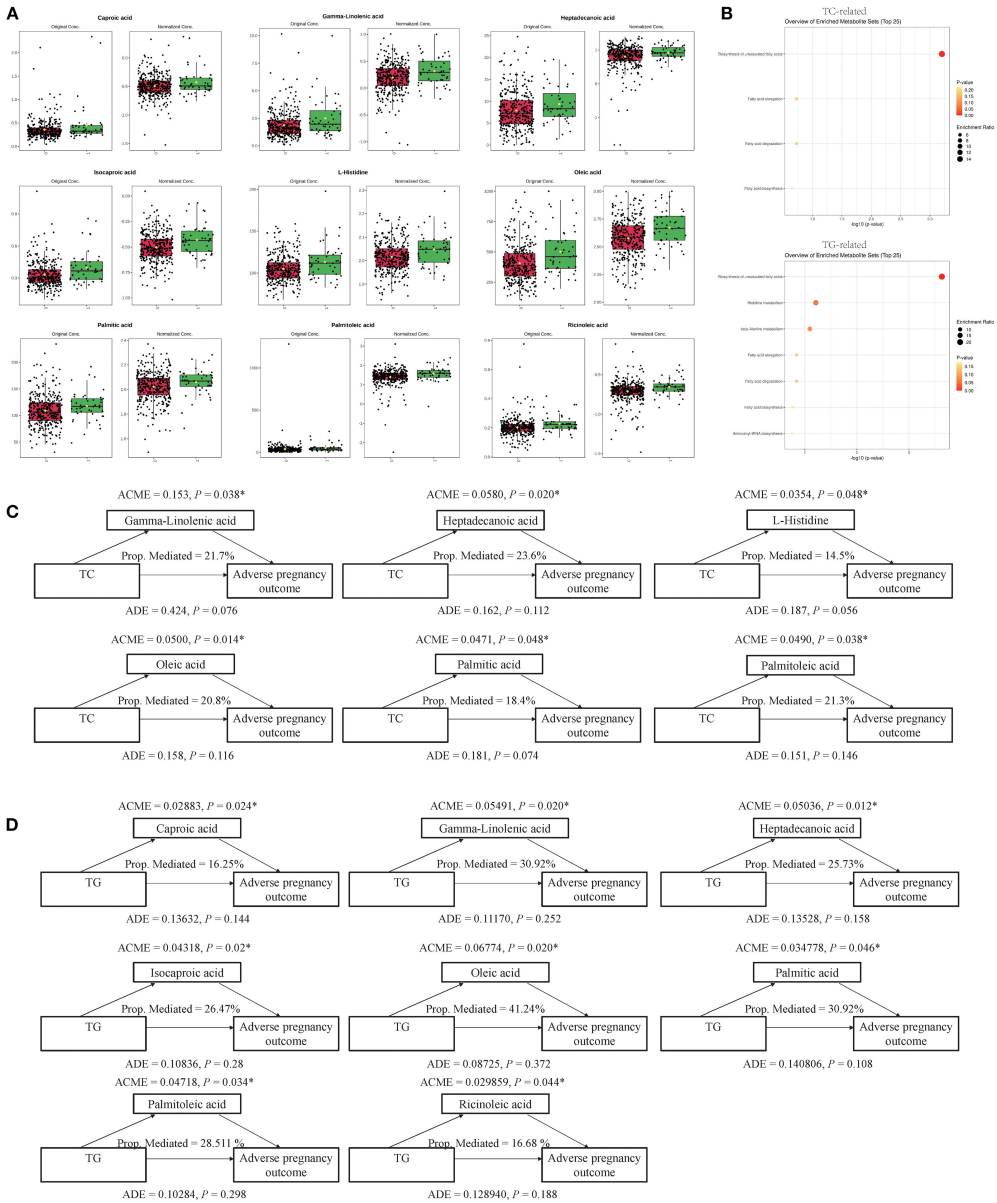
Key metabolites and their mediation effects on the causal relationship between clinical serum lipids and adverse pregnancy outcome. **(A)** Differential expression pattern of each key metabolite. **(B)** Functional enrichment of TC- and TG-related key metabolites, respectively. **(C)** Mediation models of TC and six corresponding metabolites. **(D)** Mediation models of TG and eight corresponding metabolites. *P < 0.05.

A causal mediation analysis examined whether the above metabolites mediated the association between serum lipids and adverse pregnancy outcomes, and we obtained nine key metabolic markers (Figure 2A). Among the 22 metabolites, we observed significant mediating effects of six key metabolic markers (gamma-linolenic acid, heptadecanoic acid, L-histidine, oleic acid, palmitic acid, and palmitoleic acid) on the association between TC and adverse pregnancy outcomes (*P* < 0.05, mediated proportion = 14.5–23.6%) (Figure 2C, Supplementary Table 3). Among the 31 metabolites that were significantly associated with both, TG and adverse pregnancy outcomes, eight key metabolic markers (caproic acid, gamma-linolenic acid, heptadecanoic acid, isocaproic acid, oleic acid, palmitic acid, palmitoleic acid, and ricinoleic acid) were found to mediate the effects of TG on adverse pregnancy outcomes with significant ACMEs (*P* < 0.05, mediated proportion = 16.25–41.24%) (Figure 2D, Supplementary Table 4). The TG- and TC-associated key metabolic markers shared five common metabolites, suggesting that serum lipids share the same metabolic molecular intermediation. Thus, these metabolite mediators would function together in the mediation network, and we further investigated the mediating effects of a group of collaborative metabolites between TC/TG and adverse pregnancy outcomes (Supplementary Tables 3, 4), and the mediation effect was still significant.

These key metabolic markers were evaluated using pathway enrichment analysis with the Kyoto Encyclopedia of Genes and Genomes (KEGG) database (Figure 2B). Among the key metabolites related to TC, the biosynthesis of unsaturated fatty acid metabolism pathways had the highest fold enrichment, with *P* < 0.05 and FDR <0.05 (Table 4 and Figure 2B). Similar results were obtained for key metabolites associated with TG (Table 4 and Figure 2B). Kim et al. (22) studied the relationship between plasma fatty acids and pregnancy outcomes and found that total polyunsaturated fatty acids (PUFAs) were associated with an increased risk of abortion and stillbirth. Moreover, studies have shown that women with low levels of n-3 PUFAs (when the first double bond of PUFA occurs at the third position of the methyl end of the carbon chain) during early pregnancy are at a greater risk of preterm delivery (23). However, pregnant women with higher n-3 PUFA intake in early pregnancy are less likely to give birth to babies with SGA (24). When the ratio of n-3 PUFAs to n-6 PUFAs was studied, the results were similar; thus, longer pregnancies and higher neonatal weights were associated with higher ratios (25). Animal studies have shown that dietary supplementation with n-3 PUFAs can reduce oxidative stress in the placenta and promote the growth of fetal and placental labyrinths (26).

**Table 4:** Pathway analysis of key metabolic markers.

**Pathway name**	**Total**	**Hits**	**Raw *P***	**Holm *P***	**FDR**
TG					
Biosynthesis of unsaturated fatty acids	36	3	2.1965E-4	0.01845	0.01845
TC					
Biosynthesis of unsaturated fatty acids	36	3	1.1161E-4	0.009375	0.009375

*Pathway names with P value < 0.05 are shown in the table. Total means total number of metabolites in the metabolite set; Hits mean number of key metabolic markers in the metabolite set; Raw P refers to the original P-value in the pathway analysis; Holm P refers to adjusted raw P-value by the Holm-Bonferroni method; FDR is the false discovery rate*.

### Pathway and biological function enrichment of key marker-related metabolic communities

**Figure 3:**
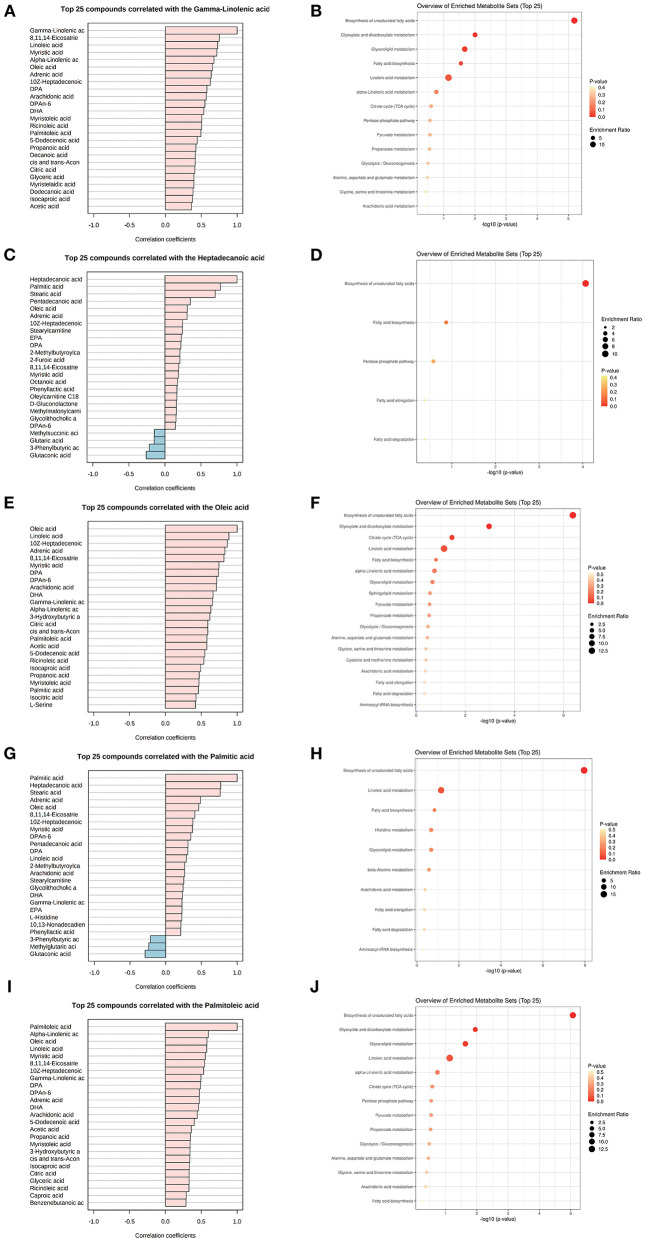
Metabolite community of common key metabolites playing mediation roles with TC and TG. List of metabolites associated with a key metabolite, gamma-linolenic acid **(A,B)**, heptadecanoic acid **(C,D)**, oleic acid **(E,F)**, palmitic acid **(G,H)**, and palmitoleic acid **(I,J)** in the metabolomics co-expression network, and their enriched KEGG functions.

The five common key metabolic markers (gamma-linolenic acid, heptadecanoic acid, oleic acid, palmitic acid, and palmitoleic acid) were differentially expressed, and their related metabolic communities were further analyzed by co-expression network analysis. Their enriched biological functions were also evaluated using pathway enrichment analysis with KEGG (Supplementary Table 5 and Figure 3). For gamma-linolenic acid-, heptadecanoic acid-, palmitoleic acid-, and palmitic acid-related metabolic communities, the biosynthesis of the unsaturated fatty acids pathway had the highest fold enrichment, with FDR <0.05. Additionally, oleic acid-related metabolic communities are primarily involved in the biosynthesis of unsaturated fatty acids and glyoxylate and dicarboxylate metabolism pathways. Our results suggest that, in addition to the biosynthesis of unsaturated fatty acids, other glyoxylate and dicarboxylate metabolism pathways should be studied. Glyoxylate and dicarboxylate metabolism are involved in carbohydrate metabolism. In a previous study, glyoxylate and dicarboxylate metabolism were significantly altered in maternal serum of those with pre-gestational(PGDM) or GDM compared with controls throughout pregnancy (27). Animal studies have shown that high glucose intake can significantly stimulate hypertriglyceridemia in Dahl salt-sensitive rats (similar to prediabetes) and lower serum TC levels. Furthermore, metabolic pathway analysis showed that high glucose intake interfered with glyoxylic and dicarboxylic acid metabolism (28).

## Discussion

First, logistic regression analysis showed that TG and TC levels were independent risk factors for adverse pregnancy outcomes in patients with GDM. In fact, from the twelfth week of gestation, blood lipid profiles, including cholesterol (TC, LDL-C, and HDL-C), and TG levels, were elevated due to estrogen stimulation and insulin resistance (29, 30). In a study conducted in China (31), women with GDM had higher concentrations of TG (2.05 ± 0.97 vs. 2.38 ± 1.37 mmol/L, *P* = 0.001) and lower levels of HDL-C (2.02 ± 0.41 vs. 1.86 ± 0.42 mmol/L, *P* < 0.001) than those in the control group. However, TC, LDL-C, ApoA1, and ApoB were not significantly different between the two groups. Moreover, in a systematic review and meta-analysis (32), TG levels were found to be significantly higher in women with GDM in each trimester of pregnancy than in the control group. Additionally, TC was higher and HDL-C was lower in the first and second trimesters of pregnancy in women with GDM than in healthy women.

Our findings were consistent with previous studies showing that maternal TG levels were positively associated with the risk for LGA newborns independent of glycemic control in women with GDM (15, 33). Another study (34) demonstrated that the TG/HDL-C ratio in the second trimester was associated with the risk of GDM (OR = 1.64, *P* = 0.02) and LGA (OR = 2.87, *P* < 0.01), and was significantly higher in pregnant women who gave birth to babies with macrosomia, which may be related to insulin resistance (35). Moreover, a matched cohort study (36) found that a serum ApoB level > 4.04 g/L combined with a TG/HDL-C ratio > 1.36 could predict the occurrence of macrosomia in the GDM and normal glucose tolerance (NGT) groups. Mediation analysis showed that ApoB and the TG/HDL-C ratio mediated the harmful effects of FBG on the risk of macrosomia.

This study also found that maternal TC levels were associated with adverse neonatal outcomes. In a review and meta-analysis (37), maternal high TG and low HDL-C levels during pregnancy were associated with increased birth weight, a higher risk of LGA and macrosomia, and a lower risk of SGA, especially in women who were overweight or obese before pregnancy. Additionally, maternal TC levels throughout pregnancy were positively associated with a small increase in birth weight but not with a higher risk of LGA and macrosomia. There may be several reasons for these inconsistent findings. For example, in those studies they did not confine the effect of confounding factors on GDM and lipid profiles. Additionally, these studies used different kits and methods to detect lipid profiles and different criteria for diagnosing GDM.

The UPLC-MS/MS metabolomics method was used to analyze the DEMs between patients with GDM with normal pregnancy outcomes and those with poor pregnancy outcomes using serum samples from the second or third trimester of pregnancy. Changes in serum metabolites were further investigated using logistic regression analysis and a general linear model, and mediation models were used to verify the association between TG or TC and adverse pregnancy outcomes through metabolites. Eight and six metabolite biomarkers were associated with TG or TC and adverse pregnancy outcomes, respectively. Five of these were identical, all of which were fatty acids.

Fatty acids include saturated fatty acids (SFAs) and unsaturated fatty acids, which are further divided into monounsaturated fatty acids (MUFAs) and polyunsaturated fatty acids (PUFAs). The circulating concentrations of even-chain SFAs [myristic acid (14:0), palmitic acid (16:0), and stearic acid (18:0)] reflect both exogenous intake (via dietary) and endogenous synthesis (via de novo lipogenesis) (38, 39), whereas odd-chain SFAs [pentadecanoic acid (15:0) and heptadecanoic acid (17:0)] mainly reflect the intake of dairy fats in the diet (40).

Palmitic acid contains 16 carbon atoms and is the most abundant saturated fatty acid in the human body. Numerous studies have found that palmitic acid levels are elevated in women prior to, at the same time as, or after GDM diagnosis (41). However, studies on palmitic acid and adverse outcomes of GDM are limited. In our study, the palmitic acid concentration was elevated in the A group, which is biologically plausible. Palmitic acid can impair the insulin signaling pathway, IRS/P13K/Akt, promoting IR, IRS-1, and Akt ubiquitination and subsequent protein degradation, leading to insulin resistance (42). Palmitic acid acts synergistically with lipopolysaccharide to activate toll-like receptor 4 (TLR4), which binds to the corresponding ligand to further activate the nuclear factor kappa-B (NF-κB) and mitogen-activated protein (MAPK) signaling pathways to promote expression of IL-6 and other inflammatory cytokines (43). Furthermore, islet beta cells are rich in endoplasmic reticulum (ER), which folds, transports, and processes insulin. Therefore, ER stress is an important pathological mechanism underlying islet β-cell dysfunction and insulin resistance. Accumulation of palmitic acid metabolites can lead to lipid apoptosis in islet β-cells by inducing ER stress (44). Palmitic acid was found to be positively correlated with HOMA-IR and markers of hyperlipidemia (e.g., TC, LDL-C, and TC), and inversely correlated with adiponectin, which in turn was associated with impaired glucose tolerance and insulin homeostasis (45). We hypothesized that triglycerides and cholesterol contribute to adverse pregnancy outcomes of GDM through palmitic acid.

Heptadecanoic acid is an odd-chain SFA. Studies of heptadecanoic acid and GDM are rare. However, previous studies have shown that heptadecanoic acid levels are elevated in women with GDM (41). The limited experimental data on heptadecanoic acid suggest that it may exert protective effects on glucose homeostasis by inhibiting hepatic oxidation. Since odd-chain SFAs mainly reflect the dietary intake of dairy fats, their (40) role in GDM and adverse pregnancy outcomes may be more affected by diet, which should be considered in future studies.

Both palmitoleic acid and oleic acid are MUFAs. Palmitoleic acid has two isoforms: cis (16:1c9) and trans (16:1t9) palmitoleic acid. The cis isoform (cis-palmitoleic acid) originates from *de novo* lipogenesis, while trans-palmitoleic acid is mainly found in dairy products and partly in hydrogenated oil. Lipogenesis is mediated by stearoyl COA desaturase 1 (SCD1), a rate-limiting enzyme for the synthesis of MUFAs, mainly oleic acid and palmitoleic acid (46). In humans, cis-palmitoleic acid biosynthesis mainly occurs in the liver, followed by adipose tissue. Moreover, plasma palmitoleic acid concentration was found to be positively correlated with the self-reported intake of whole milk products, butter, margarine, and baked desserts (47). Studies in humans have revealed a direct link between carbohydrate intake and plasma palmitoleic acid, indicating upregulation of *de novo* adipogenesis (48). In addition to carbohydrate intake, protein intake is also associated with plasma palmitoleic acid (49). Therefore, although there is evidence that some dietary components can change the concentration of palmitoleic acid, further studies on nutrient distribution and specific nutrients are needed to clarify whether dietary and lifestyle changes induce metabolic improvement through palmitoleic acid (50). Palmitoleic acid is relatively more abundant in adipose tissue than in serum. In most studies, palmitoleic acid abundance in cholesterol esters was associated with insulin sensitivity (51, 52). Only two early studies on the association between palmitoleic acid in adipose tissue and insulin resistance in adult men used a clamp for evaluation (53, 54). Endogenously produced or dietary palmitoleic acid reduces the onset of diabetes (50). Related research on palmitoleic acid and the adverse outcomes of GDM is lacking; however, previous studies have reported higher levels of palmitoleic acid in women with GDM (41). Our study also showed upregulation of palmitoleic acid in women with GDM with adverse pregnancy outcomes. However, the possible mechanism is difficult to elucidate. Further research is needed to clarify the effects of palmitoleic acid on adverse outcomes associated with GDM and whether the application of palmitoleic acid could reduce the risk of adverse outcomes.

Oleic acid (18:1) is an important MUFA; however, it is not an essential fatty acid and can be synthesized in the human body. Most studies on oleic acid have focused on the importance and possible beneficial effects of MUFA in the diet. Cortes et al. showed that a diet rich in MUFAs can reduce postprandial monocyte inflammation associated with metabolic syndrome (55). MUFAs also have beneficial effects on insulin sensitivity and type 2 diabetes mellitus (56). In addition, the reduction of SFAs and simultaneous addition of MUFAs to the diet can improve insulin sensitivity (57). Few studies have explored oleic acid and its associated adverse pregnancy consequences. Further studies are needed to explore the effects and possible mechanisms of oleic acid on maternal outcomes.

There are two isoforms of linolenic acid (LA): alpha-linolenic acid (ALA) and gamma-linolenic acid (GLA), also known as omega-3 and omega-6, respectively. GLA is an n-6 PUFA that can be derived from both exogenous (through dietary intake) and endogenous (through lipogenesis) sources. A previous study indicated that the major endogenous metabolism of plasma phospholipid n-6 PUFAs, including GLA and DGLA, has a potential role in the development of GDM during the first to second trimesters of pregnancy (58). Studies have shown that patients with GDM have a unique fatty acid metabolism profile consisting of increased n-6 PUFA levels, decreased n-3 PUFA levels, and abnormal n-6 PUFA metabolism. Previous studies have shown that the dietary intake of LA has little effect on the circulation of GLA and DGLA, indicating that LA has a strong endogenous regulatory effect on these metabolites (59). However, a recent study showed no association between LA and markers of glucose homeostasis and risk of GDM, suggesting that circulating LA does not play a harmful role in GDM pathophysiology (58). Further investigations on the role of GLA and adverse pregnancy outcomes in patients with GDM are warranted.

Finally, according to metabolic enrichment and pathway analyses, biosynthesis of unsaturated fatty acids was consistently found to be the key metabolic pathway in both TG- and TC-related metabolites, indicating that the metabolism of abnormal fatty acids is a major mechanism in poor pregnancy outcomes of GDM.

Our study further proved that TG and TC were independent risk factors for adverse pregnancy outcomes in patients with GDM, and confirmed that lipids can influence adverse pregnancy outcomes in GDM through serum metabolites. The mediator metabolites would mainly participate in the biosynthesis of unsaturated fatty acids pathway, which may shed new light on the pathology of GDM and provide insights for further exploration of the molecular mechanisms underlying adverse pregnancy outcomes. However, this study also has some shortcomings. First, the number of participants included was not very large, which may affect the effectiveness of our statistical analysis. Therefore, a larger group of participants is needed to be verified the conclusions of this study. Second, the exact molecular mechanism underlying the GDM adverse pregnancy outcomes is unclear, and further research is needed to clarify the exact role of these found metabolites in GDM.

## Conclusion

In conclusion, our study demonstrated the mediating role of metabolomic profiles between clinical lipids and adverse pregnancy outcomes of GDM using omics analysis of serum samples. This study suggests that a specific metabolomic profile exists between GDM lipid levels and adverse pregnancy outcomes. Several key metabolites, such as gamma-linolenic acid, heptadecanoic acid, oleic acid, palmitic acid, and palmitoleic acid, have been identified as potential biomarkers for adverse pregnancy outcomes in patients with GDM. These metabolites mainly participate in the biosynthesis of unsaturated fatty acids. In the future, further research should be conducted to confirm our findings and explore the underlying molecular mechanisms by expanding the cohort size and lifestyle factors.

## Data availability statement

The datasets presented in this article are not readily available because informed consent does not contain data that can be disclosed to third parties. Requests to access the datasets should be directed to CH, alfredhc@sjtu.edu.cn.

## Ethics statement

The studies involving human participants were reviewed and approved by the Ethics Committee of the University of Hong Kong-Shenzhen Hospital ([2014] 98, [2017] 13). The patients/participants provided their written informed consent to participate in this study.

## Author contributions

CH and XY supervised this study. ML, MT, WLu, QL, and XF collected and checked data. CH and ML designed the experiments and analyzed the data. RZ, XF, WLu, and ML helped carry out the experiments. XY, ML, and JG performed the data analysis. ML, JG, WLi, and XY prepared and revised the manuscript. All authors have read and approved the final manuscript.

## Funding

This study was supported by the High-Level Hospital Program, Health Commission of Guangdong Province, China (HKUSZH201901025), National Natural Science Foundation of China (No. 81974118), Shanghai Science and Technology Innovation Action Plan (Grant No. 20XD1433300), and Shanghai Jiao Tong University Affiliated Sixth People's Hospital Basic Scientific Research (No. ynms202118).

## Conflict of interest

The authors declare that the research was conducted in the absence of any commercial or financial relationships that could be construed as a potential conflict of interest. The handling editor FD declared a past co-authorship with the author CH.

## Publisher's note

All claims expressed in this article are solely those of the authors and do not necessarily represent those of their affiliated organizations, or those of the publisher, the editors and the reviewers. Any product that may be evaluated in this article, or claim that may be made by its manufacturer, is not guaranteed or endorsed by the publisher.
