# Supplementary material for: The mediating role of maternal metabolites between lipids and adverse pregnancy outcomes of gestational diabetes mellitus
Source: Front Med (Lausanne). 2022 Aug 10;9:925602. doi: 10.3389/fmed.2022.925602 (PMC9400014; doi:10.3389/fmed.2022.925602)
Supplement: Supplementary file 1 [file Table_3.docx]

Table S3 Mediation models of the association between TC and adverse pregnancy outcomes through metabolites.

| Metabolites | ACME (95% CI) | *P* | Mediated proportion (%) |
| --- | --- | --- | --- |
| Adrenic acid | 0.02221 (-0.02856-0.08) | 0.28 | - |
| Alpha-Linolenic acid | 0.03440 (-0.00768-0.08) | 0.12 | - |
| Arachidonic acid | 0.03302 (-0.02236-0.10) | 0.18 | - |
| Gamma-Linolenic acid | 1.53e-01 (5.89e-03-0.40) | 0.038* | 21.7 |
| Heptadecanoic acid | 5.80e-02  (8.03e-03-0.13) | 0.020* | 23.6 |
| Indoleacrylic acid | -0.03262 (-0.09317-0.01) | 0.18 | - |
| Isocaproic acid | 4.74e-02 (-1.06e-03-0.11) | 0.06 | - |
| L-Arginine | 0.03553 (-0.02650-0.08) | 0.24 | - |
| L-Glutamine | 0.01971 (-0.00826-0.05) | 0.18 | - |
| L-Histidine | 3.54e-02 (1.55e-04-0.09) | 0.048* | 14.5 |
| L-Homoserine | 0.02280 (-0.00989-0.06) | 0.12 | - |
| L-Lactic acid | 0.01965 (-0.02565-0.06) | 0.32 | - |
| L-Serine | 0.02147  (-0.00798-0.07) | 0.22 | - |
| L-Threonine | 2.19e-02 (-1.04e-03-0.05) | 0.08 | - |
| Linoleic acid | 0.042255 (-0.003764-0.09) | 0.08 | - |
| Myristic acid | 0.05250 (-0.01045-0.10) | 0.12 | - |
| Oleic acid | 5.00e-02 (7.26e-03-0.11) | 0.014* | 20.8 |
| Oxoglutaric acid | 0.01397 (-0.01296-0.05) | 0.40 | - |
| Palmitic acid | 4.71e-02 (1.95e-04-0.11) | 0.048* | 18.4 |
| Palmitoleic acid | 4.90e-02 (3.04e-03-0.11) | 0.038* | 21.3 |
| 8,11,14-Eicosatrienoic acid | 0.04020 (-0.01294-0.11) | 0.18 | - |
| 10Z-Heptadecenoic acid | 0.053975 (-0.011129-0.14) | 0.08 | - |
| Metabolite score | 0.08728  (0.03232-0.17) | <0.001* | 32.85 |

The metabolite score was calculated by a weighted sum of concentrations of 6 significant metabolites.

Table S4 Mediation models of the association between TG and adverse pregnancy outcomes through metabolites.

| Metabolites | ACME (95% CI) | *P* | Mediated proportion (%) |
| --- | --- | --- | --- |
| Adrenic acid | 0.02394  (-0.02928-0.07) | 0.34 | - |
| Alpha-Linolenic acid | 0.04309 (-0.00922-0.10) | 0.12 | - |
| Arachidonic acid | 0.0254  (-0.0201-0.07) | 0.22 | - |
| Benzenebutanoic acid | 0.02554  (-0.01105-0.07) | 0.10 | - |
| Caproic acid | 0.02883 (0.00286-0.07) | 0.024* | 16.250 |
| Citric acid | 0.0517 (-0.0208-0.12) | 0.16 | - |
| Gamma-Linolenic acid | 0.05491 (0.00915-0.11) | 0.020* | 30.916 |
| Glucaric acid | 3.88e-02 (-2.47e-03-0.11) | 0.10 | - |
| Glyceric acid | 0.01224 (-0.01846-0.05) | 0.44 | - |
| Heptadecanoic acid | 0.05036 (0.00863-0.11) | 0.012* | 25.730 |
| Isocaproic acid | 0.04318 (0.00604-0.09) | 0.02* | 26.472 |
| Isocitric acid | 0.0393 (-0.0418-0.12) | 0.28 | - |
| L-Arginine | 0.03492 (-0.00907-0.09) | 0.20 | - |
| L-Histidine | 0.02278 (-0.00162-0.06) | 0.12 | - |
| L-Homoserine | 0.01369 (-0.00925-0.05) | 0.34 | - |
| L-Lactic acid | 0.00946 (-0.00864-0.04) | 0.28 | - |
| L-Serine | 0.02157 (-0.01612-0.06) | 0.34 | - |
| L-Threonine | 0.02004 (-0.00485-0.06) | 0.16 | - |
| Linoleic acid | 6.27e-02 (2.25e-05-0.14) | 0.06 | - |
| Myristic acid | 0.06324 (-0.00848-0.13) | 0.08 | - |
| N-Acetylglycine | 0.029739 (-0.008165-0.08) | 0.12 | - |
| Oleic acid | 0.06774 (0.00884-0.13) | 0.020* | 41.237 |
| Oxoglutaric acid | 0.01388 (-0.00500-0.04) | 0.18 | - |
| Palmitic acid | 0.034778 (0.001368-0.08) | 0.046* | 19.1621 |
| Palmitoleic acid | 0.04718 (0.00300-0.11) | 0.034* | 28.511 |
| Propanoic acid | 0.01653 (-0.01983-0.06) | 0.44 | - |
| Ricinoleic acid | 0.029859 (0.000157-0.07) | 0.044* | 16.6854 |
| 8,11,14-Eicosatrienoic acid | 0.04498 (-0.01808-0.100) | 0.16 | - |
| cis and trans-Aconitic acid | 0.04121 (-0.04407-0.12) | 0.26 | - |
| 10Z-Heptadecenoic acid | 0.051118 (-0.000797-0.12) | 0.08 | - |
| 3-Hydroxybutyric acid | 0.01336 (-0.01283-0.04) | 0.32 | - |
| Metabolite score | 0.09954  (0.04340-0.17) | <0.001* | 51.29 |

The metabolite score was calculated by a weighted sum of concentrations of 8 significant metabolites.

Table S5 Pathway analysis of key marker related metabolic communities

| Key marker | Pathway name | Total | Hits | Raw P | Holm P | FDR |
| --- | --- | --- | --- | --- | --- | --- |
| gamma-linolenic acid | Biosynthesis of unsaturated fatty acids | 36 | 6 | 2.23E-06 | 0.00018703 | **0.00018703** |
|  | Glyoxylate and dicarboxylate metabolism | 32 | 3 | 0.0061909 | 0.51385 | 0.26002 |
|  | Glycerolipid metabolism | 16 | 2 | 0.015427 | 1 | 0.37822 |
|  | Fatty acid biosynthesis | 47 | 3 | 0.018011 | 1 | 0.37822 |
| heptadecanoic acid | Biosynthesis of unsaturated fatty acids | 36 | 5 | 1.85E-05 | 0.001551 | **0.001551** |
| oleic acid | Biosynthesis of unsaturated fatty acids | 36 | 7 | 1.24E-07 | 1.04E-05 | **1.04E-05** |
|  | Glyoxylate and dicarboxylate metabolism | 32 | 4 | 0.000576 | 0.047798 | **0.024187** |
|  | Citrate cycle (TCA cycle) | 20 | 2 | 0.026157 | 1 | 0.73239 |
| palmitic acid | Biosynthesis of unsaturated fatty acids | 36 | 8 | 7.83E-10 | 6.58E-08 | **6.58E-08** |
| palmitoleic acid | Biosynthesis of unsaturated fatty acids | 36 | 6 | 2.23E-06 | 0.000187 | **0.000187** |
|  | Glyoxylate and dicarboxylate metabolism | 32 | 3 | 0.006191 | 0.51385 | 0.26002 |
|  | Glycerolipid metabolism | 16 | 2 | 0.015427 | 1 | 0.43195 |

Pathway name with P value < 0.05 are shown in the table. Total means total number of metabolites in the metabolite set; Hits mean number of key metabolic markers in the metabolite set; Raw P refers to original P value in the pathway analysis; Holm P refers to adjusted raw P value by Holm-Bonferroni method; FDR is the false discovery rate.
